# Executive Functions in Parkinson’s disease with and without Deep Brain Stimulation (DBS): A systematic review

**DOI:** 10.1590/1980-57642020dn14-020012

**Published:** 2020

**Authors:** Thayná Laís de Souza Arten, Amer Cavalheiro Hamdan

**Affiliations:** 1 Master in Psychology, Department of Psychology, Federal University of Paraná, Curitiba, PR, Brazil.; 2 PhD, Department of Psychology, Federal University of Paraná, Curitiba, PR, Brazil.

**Keywords:** Parkinson’s Disease, Deep Brain Stimulation, Executive Functions, Neuropsychological Assessment, doença de Parkinson, estimulação cerebral profunda, funções executivas, avaliação neuropsicológica

## Abstract

Given the aging of the world population, it is essential to investigate which methods are most effective for treating the diseases that manifest with age. This study addresses Parkinson’s disease (PD), for which Deep Brain Stimulation (DBS) has been increasingly used to treat the disease.

**Objective:**

To investigate Executive Functions (EF) in patients with and without DBS.

**Methods:**

A systematic review of the literature was conducted according to the Preferred Reporting Items for Systematic Review and Meta-Analyzes (PRISMA) criteria. Scientific papers published on the Scopus, Web of Science and PsycInfo databases were selected.

**Results:**

13 articles were selected. Results showed no standardization of instruments used to evaluate EF and that, in most studies, lack of a control group may have affected results.

**Conclusion:**

Decline in EF was observed in terms of verbal fluency and processing speed in patients with DBS.

## RESUMO.

Observando o envelhecimento da população mundial, é essencial investigar quais métodos são mais eficazes no tratamento das doenças que aparecem com a idade. Este estudo aborda a doença de Parkinson (DP). Na DP tem aumentado o uso da Estimulação Cerebral Profunda (DBS) para o tratamento da doença.

**Objetivo:**

Investigar a Função Executiva (FE) em pacientes com e sem DBS.

**Métodos:**

Foi realizada uma revisão sistemática da literatura de acordo com os critérios de Principais Itens para relatar Revisão Sistemática e Meta-Análises (PRISMA). Selecionamos artigos científicos publicados nas bases de dados Scopus, Web of Science e PsycInfo.

**Resultados:**

13 artigos foram selecionados. Observou-se que não há padronização nos instrumentos utilizados para avaliar a FE e, na maioria dos estudos, a falta de controle de grupo pode ter afetado os resultados.

**Conclusão:**

Observou-se declínio na FE na fluência verbal e velocidade de processamento em pacientes com DBS.

According to data from World Population Prospects: the 2019 Revision, by 2050, one in six people in the world will be over age 65 (16%), up from one in 11 in 2019 (9%). In 2018, for the first time in history, persons aged 65 or above outnumbered children under five years of age globally. The number of persons aged 80 years or over is projected to triple, from 143 million in 2019 to 426 million in 2050.^1^ This growth will be accompanied by a commensurate increase in Parkinson’s disease (PD) and other neurodegenerative problems, where PD is one of the most common movement disorders and neurodegenerative disease in the elderly, affecting Executive Functions.^2^

Studying the possible effects of DBS implantation on Executive Functions in Parkinson’s patients is important because Executive Dysfunctions (ED) are the basis for manifestations of cognitive impairment in these patients (due to disruption of striatal dopamine flow). In addition, EF can also be affected by changes such as depression, which is the most frequent mood disorder in PD.^2^

PD was initially described by James Parkinson as “Agitating Paralysis” in 1817.^3^ Several drug therapies have been applied since 1867 in the treatment of PD, with dopamine replacement being the most common.^3^ However, due to the therapeutic limitations of available treatments, many studies are being conducted to find alternatives for the treatment of PD. One of these alternatives is the surgical intervention of Deep Brain Stimulation (DBS) implantation. Given this entails brain surgery, it is necessary to determine possible changes caused by the intervention in patient cognition and possible impact on their daily lives. It should be noted that the DBS implantation procedure is only recommended when the patient is refractory to medications.^4^

Neurons are structures known to be susceptible to variations in the electrical potential of their cell membrane when exposed to a variable electric field. The DBS device activates brain structures through implanted electrodes and is used to treat neurological and psychological disorders, often reducing ineffective medication administration.^5^ It is common to implant DBS electrodes for Parkinson’s disease in the Subthalamic Nucleus (STN), and for psychological problems in the Internal Globus Pallidus (GPi). The surgical procedure is performed using stereotactic neurosurgical techniques, with local anesthesia and with the patient awake. After electrode implantation, the pulse generator (similar to a cardiac pacemaker) is placed within the patient, but under general anesthesia, and usually in the subclavicular or chest region, but can also be placed directly in the skull.^5^

In view of the growing use of surgical interventions as a treatment for PD, we sought to understand how these interventions affect patient cognition and possible impact on their daily lives, analyzing the results of previous studies conducted with these patients in the global literature. The aim of this systematic review was to analyze empirical studies, considering that the results on the subject are controversial and the fact that there is almost no research comparing ways to treat Parkinson’s disease and its effects on executive functions (no publications found in the databases searched).

## METHODS

A systematic review of the literature was conducted according to the Preferred Reporting Items for Systematic Review and Meta-Analyzes (PRISMA)^6^ criteria. The following terms were used: “Deep Brain Stimulation”, “Parkinson Disease” and “Executive Functions” with the Boolean operator “AND”. Scientific papers published in all languages without delimiting a period, involving comparative clinical trials in humans, were selected from the Scopus, Web of Science and PsycInfo databases. The inclusion criteria were a neuropsychological assessment of Parkinson’s patients with and without DBS, comparison between Parkinson’s and control groups, and use of classic neuropsychological tests. The exclusion criteria were studies of systematic reviews, case studies, book chapters, absence of a neuropsychological assessment and of inclusion and exclusion criteria, and focus on aspects other than Executive Functions.

### Study selection

**Figure 1:**
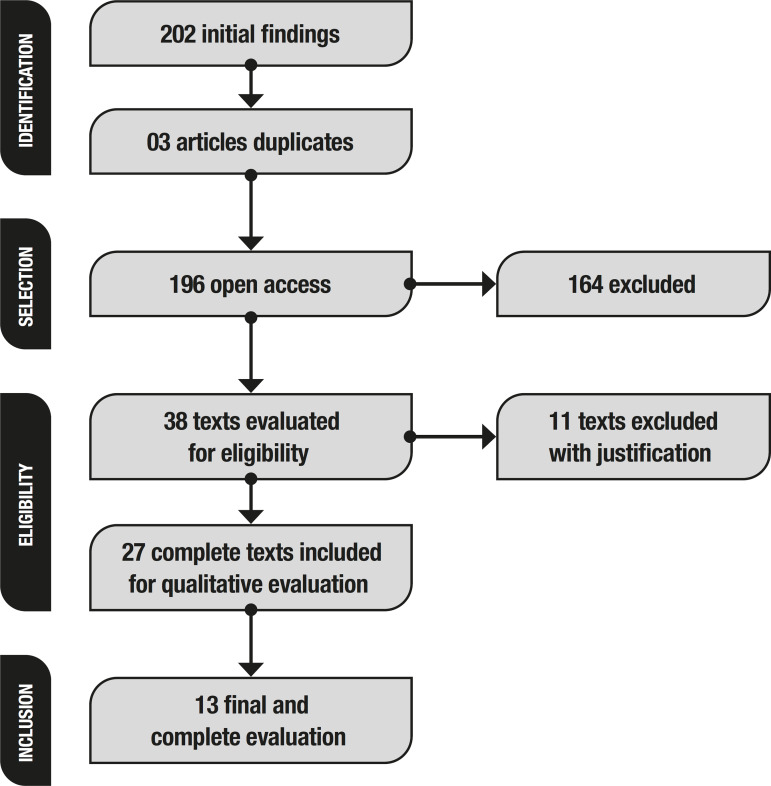
Literature search flow diagram.

Initially, this method retrieved 202 studies (Figure 1). A total of 22 articles was found on the PsycInfo database, but 2 were later excluded because of access difficulties. Five articles were found on the Web of Science database, 1 of which could not be accessed. On the Scopus database, 175 articles were found. To refine the search, a filter was added in “Field of Research: Psychology” because the focus of this study was to determine, through neuropsychological assessment, differences between the interventions for PD,. This led to retrieval of 21 articles , of which 17 were published in journals, and only 14 accessible.

Based on publications retrieved, titles and abstracts were examined for studies involving only human clinical trials and applying a neuropsychological assessment, excluding duplicates, review studies, case studies, meta-analyses, and those related only to the field of medicine, giving a total of 27 studies for analysis. After full reading of these articles, those with problems in the methodology were excluded, giving a final total of 13 studies for comparison. The researchers selected the articles independently: considering suitable studies that: (a) evaluated PD patient cognition with STN-DBS; (b) reported the instruments and domains evaluated; and (c) focused on neuropsychological assessment.

## RESULTS

The final list of articles included, based on the search criteria, in order of year together with the results is given in Table 1. The list of instruments used with quantity, separated by domains and behavior measured, is given in Table 2, and the principal conclusions are outlined below. A total of 40 (forty) instruments were used to evaluate different aspects of patients, including batteries, subtests, scales and tasks (Table 2). These can be ordered from the most evaluated to least used, as follows: executive functions, memory, global cognitive functioning, language, mood, visuoconstructive skills, and attention.

**Table 1:** List of articles included in the systematic review criteria.

Authors and year	Sample	Neuropsychological Instruments used	Location
Ceravolo, R. et al. 2011^14^	**6 total DBS group**	California Verbal Learning Test; Trail Making Test (TMT); Verbal Fluency (FAS); Boston naming test.	Subthalamic Nucleus DBS (bilateral) - STN
Obeso, I. et al. 2012^15^	217 (male) 83 (female) **300 total PD group** 22 (male) 28 (female) **50 total control group**	Frontal Assessment Battery (FAB); Verbal Fluency (letter F)	No DBS used
Fumagalli, M. et al. 2015^16^	5 (male) 6 (female) **11 total DBS group** 5 (male) 6 (female) **11 total PD group**	Digit Span; Corsi Block-Tapping test; Babcock Story Recall Test; Raven Colored Progressive Matrices; FAB; Phonological and Semantic Verbal Fluency; Attentional Matrices; Constructional Apraxia Test.	Subthalamic Nucleus DBS (bilateral) - STN
Tang, V. et al. 2015^17^	18 (male) 9 (female) **27 total DBS group**	MoCa; Chinese Auditory Verbal Learning Test (CAVLT); Benton Visual Retention Test (BVRT); a Chinese version of the Boston naming test (BNT); Hooper visual organization test (HVOT); digit span test (DST); Stroop test Chinese Victoria version; Verbal Fluency test.	Subthalamic Nucleus DBS (bilateral) - STN
Houvenaghel, JF. et al. 2016^18^	10 (male) 15 (female) **25 total PD group** 12 (male) 13 (female) **25 total DBS group**	Verbal Fluency (animals) and Phonemic Fluency (letter p); Nelson's simplified version of the Wisconsin Card Sorting Test (MCST); Trail Making Test; Stroop Test.	Subthalamic Nucleus DBS (bilateral) - STN
Foley, J. et al. 2017^19^	17 (male) 11 (female) **28 total DBS group**	Stroop; Hayling Sentence Completion Test; Brixton Spatial Anticipation Test; Elevator Counting and Distraction subtests from the Test of Everyday Attention (EC, EC-D); Nelson Modified Card Sorting Test (MCST), and Trail Making Test (TMT-B/A); Symbol Search (SS) and Digit Symbol Coding (DSC) of WAIS-III.	Subthalamic Nucleus DBS (bilateral) - STN
Ardouin, C. et al. 1999^7^	49 STN DBS: 24 (male), 25 (female) 13 GPi: 9 (male), 4 (female) **62 total DBS group**	Wisconsin Card Sorting Test; Verbal Fluency (fruits or furniture); Literal Fluency (letter V or R); Graphic and motor series; Stroop Test; Trail Making Test.	Subthalamic Nucleus DBS (bilateral) - STN Globus pallidus internal - GPi
Witt, K. et al. 2004^8^	17 (male) 6 (female) **23 total DBS group**	Digit Span (forward and backward); Verbal Fluency (female/male, first name, animals/plants); Literal Fluency (K/N or L/M), Stroop, Random Number Generation Task (RNGT).	Subthalamic Nucleus DBS (bilateral) - STN
Funkiewiez, A. et al. 2004^9^	43 (male) 34 (female) **77 total DBS group**	Wisconsin card sorting test (WCST); Category and Literal Fluency; Graphic and Motor Series; Grober and Buschke test.	Subthalamic Nucleus DBS (bilateral) - STN
Castelli, L. et al. 2006^10^	38 (male) 27 (female) **65 total DBS group**	Raven Color Matrices; Bi-syllabic Word Repetition Test (BWR); Corsi's Block-Tapping Test (CBT); Paired- Associate Learning (PAL), a Wechsler Memory Scale subtest; Trail Making Test Part B; Nelson Modified Card Sorting Test (MCST); Wisconsin Card Sorting Test; Phonemic and category verbal fluency tasks.	Subthalamic Nucleus DBS (bilateral) - STN
Fraraccio, M. et al. 2008^11^	9 (male) 6 (female) **15 total DBS group**	Rey Auditory Verbal Memory Test; Wechsler Memory Scale; Rey Osterreith Figure/Taylor Figure, WAIS-Digit Span (forward and backward); Tower of London; Wisconsin Card Sorting Test; Stroop; Symbol Digital Modalities Test; Hooper Visual Organizational Test; Rey Figure/Taylor Figure; Boston Naming Test; Controlled Oral Word Association Test.	Subthalamic Nucleus DBS (bilateral) - STN
Mikos, A. et al. 2009^12^	12 (male) 7 (female) **19 total PD group** 20 (male) 4 (female) **24 total DBS group** **43 total sample**	Verbal Fluency (Word and Animals); Digit Span Backward; Boston Naming Test; Hopkins Verbal Learning Test-Revised (HVLT-R), Logical Memory subtest of the WAIS; Trail Making Test; Stroop Test; Judgment of Line Orientation Test; Benton	Subthalamic Nucleus DBS (unilateral) - STN Right (3), left (8). Globus pallidus internal GPi Right (5), left (8)
Castelli, L. et al. 2010^13^	16 (male) 15 (female) **31 total PD group** 17 (male) 10 (female) **27 total DBS group** **58 total sample**	Raven Color Matrices; Bi-syllabic Words Repetition test; Corsi's Block Tapping; Paired Associate Learning; Trail Making B, Nelson Modified Card Sorting test, Phonemic and Category Verbal Fluency.	Subthalamic Nucleus DBS (bilateral) - STN

**Table 2:** List and frequency of instruments used.

Instruments	Used in articles
Verbal FluencyTasks -Semantic	9
Verbal Fluency Tasks - Phonemic	10
Wisconsin Cards SortingTest(WCST)	4
StroopTest	7*
Trail MakingTest(TMT)	7
Digit Span Forwardand Backward	6
Frontal AssessmentBattery (FAB)	2
Hayling SentenceCompletionTest	1
Tower of London	1
Luria graphic and motor series^18^	2
Random Number Generation Task (RNGT).	1
Nelson Modified Card Sorting Test (MCST)	4
Brixton Spatial Anticipation Test	1
Grober and Buschke test	1
Boston Naming Test (BNT)*	4*
Controlled Oral Word Association Test	1
Attentive Matrices	1
Symbol Digital Modalities	1
The judgment of Line Orientation	1
Elevator Counting and Distraction subtests from the Test of Everyday Attention (EC, EC-D);	1
Rey´s Auditory Verbal Learning Test (RAVLT)	2*

* Chinese version of the test

Of the 13 studies, only 1 had a healthy control group,^15^ 4 compared only PD *versus* DBS PD patients,^12^^,^ 13 ^,^ 16 ^,^ 18 7 were tested before and after DBS implantation or ON/OFF condition,^8^^-^ 11 ^,^ 14 ^,^ 16 ^,^ 17 ^,^ 19 and 1 compared DBS patients versus GPi patients.^7^ The review found limited use of control groups for comparing results in most studies and the absence of a specific assessment protocol for PD with and without STN-DBS (evidenced by the variability of instruments used in the different studies, 40 overall). Moreover, there was a lack of initial screening of the patient’s overall functioning, where this assesment was applied in only 5 of the 13 studies. Thus, the construction and validation of a battery for use with this population would be valuable for future studies.

Comparing the results for Executive Functions (EF) among the healthy group and PD patients, disease stage had a linear effect on Verbal Fluency that worsened with the progression of the disease. In addition, the presence of depression was associated with worsening verbal fluency score, as depression negatively influenced tasks requiring word generation, contributing to this low score. Furthermore, the authors pointed out that patients whose PD initiated in the right side of the brain had significantly worse verbal fluency than those with left-sided onset.^15^

Comparing EF between PD patients with and without DBS, a statistically significant decline in the DBS group on verbal fluency and literal semantic tests was noted, where a higher proportion of patients with DBS (50%) than controls (11%) exhibited an individual level decline in one or more structures such as fluency measures.^12^ It is important to emphasize that decline in Verbal Fluency should not be considered part of disease progression, even though patients with DBS are the most severe PD patients according to the Unified Parkinson’s Disease Rating Scale section III (UPDRS, section III), since according to the authors, this could be a side effect induced by surgery.^13^

A significant decline in Verbal Memory and delayed recall of information in the DBS group compared to the treated group was also observed.^13^ There was no group or individual level difference for the other elements tested (numbers and Boston Naming Test),^12^ but no other study or group had a higher occurrence on this task.^16^ The DBS group showed a significant increase in difficulty on the Hopkins Verbal Learning Test-Revised (HVLT-R), Trail Making Test A and B, word reading and Stroop Test, and showed the most severe motor dysfunction when evaluating the condition ‘’Off’’.^12^ In addition, the DBS group had lower Mini-Mental State Examination (MMSE) scores, which were higher in the control group, but in both groups indicated non-pathological performance.^16^ Another interesting point in the studies was when a reward was offered to the DBS group, patients proved faster and with task execution goals, suggesting that STN-DBS increased the incentive effect of promised rewards (action selection more impulsive in a low incentive context).

In general, there was a decrease in DBS groups (when compared to PD patients without DBS) on Verbal Fluency tasks and, in other cases, on immediate verbal memory and long-term memory. However, in DBS the decision-making ability in many domains is preserved and reaction times or response type do not change. DBS also influences the effect of reward incentive, when offered, thus altering the processes involved in the solution of problems. Comparing cognitive functions before and after DBS surgery, order of function was Verbal Fluency, Memory, Processing Speed, Inhibitory Control, Global Functioning, Apathy, Depression, Anxiety, and Social Aspects.

In summary: [1] Verbal Fluency - Decrease in verbal fluency test scores^8^^,^ 10 ^,^ 17 ^,^ 19 no reduction in verbal fluency after STN stimulation.^11^^,^ 14 [2] Memory - One of the studies showed no changes after STN DBS in memory or verbal learning, both of which remained stable after implantation.^10^ On the other hand, there was an improvement in the results of a verbal memory test performed 6 months after the operation when reapplied 12 months after the DBS procedure in other studies.^14^^,^ 17 [3] Processing speed -Significantly reduced after DBS, representing the most important predictor of decline in verbal fluency tests.^19^ [4] Inhibitory control - Stroop test worsened after stimulation and Random Number Generation Task (RNGT) counting after stimulation worsened in one study.^8^ These effects may be induced by stimulation of the associative territory of the STN, but there was no significant difference between groups with and without DBS.^8^ In another study, there was a significant reduction in obsessive-compulsive characteristics after STN DBS.^10^ [5] Global Functioning - There was no global deterioration in neuropsychological function attributable to STN-DBS, confirming that mild postoperative cognitive decline is transient.^9^^,^ 10 ^,^ 14 ^,^ 17 [6] Apathy - The apathy score changed significantly, showing an increasing proportion of apathetic patients over time.^9^^,^ 19 [7] Depression - Levels fluctuated, but not significantly,^9^ or remained the same, after implantation.^17^ The condition may appear with greater latency and be related to dopaminergic treatment decrease and loss of the antidepressant effect of Levodopa. However, mood was often significantly reduced postoperatively.^14^^,^ 17 [9] Social - after implantation, the motor benefit allowed some patients to be more autonomous in life, facilitating social situations.^10^

## DISCUSSION

The results found in the present study suggest that generally, after the implementation of Deep Brain Stimulation (DBS), in most studies, there was a decrease in the executive functions of Verbal Fluency, Processing Speed and some modification in apathy levels. However, the studies also suggest that there was a decrease in the degree of anxiety and, in a single case, there was an improvement in memory scores and obsessive-compulsive symptoms (related to inhibitory control).

When comparing DBS implant surgical interventions and Internal Globus Pallidus lesions, we observed an improvement in motor function for both DBS and Internal Pallidus Globe (GPi) lesion surgery. There was also a slight, significant improvement in mood, but no improvement in language, memory or other cognitive functions, indicating that the few cognitive changes detected by neuropsychological tests were subtle. Verbal fluency deficits were found after STN, but not GPi stimulation, and were not associated with executive deficits generally found in GPi lesions.^7^ These results may be related to the methodology used for patient evaluation, such as the absence of a standard evaluation protocol, and the frequent non-standardization of these instruments. Moreover, the lack of control groups in most evaluations may also have contributed to the inconclusive results (no comparison with healthy groups).

The implications of the results obtained in the literature may have a direct impact on the choice of whether to perform DBS implantation surgery. However, as the results are inconclusive about improvement/worsening or a possible impact on patient cognition, it is not possible to infer whether motor improvement offsets cognitive risk for implant candidates. Thus, further research in the area is required using more advanced evaluation methods, such as fMRI, PET, etc., in addition to traditional neuropsychological testing techniques. Thus, it would be possible to achieve more satisfactory results on the subject and thus contribute to the literature and objectively instrumentalize the choice of patients wishing to undergo surgery, despite the difficulty conducting research involving the use of neuroimaging.

Therefore, this study aimed to outline the main instruments used in patients diagnosed with PD and their frequency in studies, to know the material that has been used in the evaluation of these patients. In addition, we also sought to determine the possible cognitive effects, and their impacts on the patient’s daily life, that may appear after implementation of DBS in PD. It should be noted that, even after so many years of research on Parkinson’s disease and the use of various techniques for treating the disease, there are still no conclusions regarding the cognitive impacts of any of them, information of great importance to patients.

Considering the small number of articles on the topic of interest (Executive Functions and Parkinson’s Disease), it is necessary to interpret these data with caution. Sample sizes were small and, in 12 of the 13 cases, there was no healthy control group, precluding meaningful comparisons. In addition, the lack of a standardized battery for evaluating these patients also hampered comparison, with different instruments and modes of analysis employed.

Thus, further research should be carried out involving larger samples and healthy control groups, as well as standardized instruments to enable subsequent comparison. Moreover, longitudinal follow-up of these patients could also increase the reliability of these results.

In summary, we sought to systematize the results of studies conducted in patients diagnosed with Parkinson’s disease. Results showed that, in theory, levels of benefits and risks were equal in the implementation of the DBS. Improvements reported were attributed to memory and social life and also decreased anxiety. Worsening after intervention was observed mainly in verbal fluency, processing speed, and apathy. The other aspects assessed (inhibitory control, global development, and depression) showed no significant differences in the studies, remaining stable over time. These results highlight the need for further studies on the techniques used to treat Parkinson’s disease, given there was a noticeable decline in the cognition of these patients that may or may not be attributed to their treatment. It is necessary to clarify the cause of these declines and devise possible rehabilitation techniques and a standard assessment protocol. This protocol could start with an evaluation of the patient’s general condition, determining whether he/she has a diagnosis of depression and/or other comorbidities, the time of diagnosis in PD and surgery, main causes for the implantation. A screening instrument, such as the Montreal Cognitive Assessment (MoCA), and classic neuropsychological tests could then be applied for the evaluation of executive functions including: Rey’s Auditory-Verbal Learning Test (RAVLT) for auditory memory assessment - short and long-term; the Trail Making Test A and B for cognitive flexibility, the Semantic and Phonological Verbal Fluency Test and Stroop Test for Inhibitory Control; and the WAIS-III Digit Subtest for auditory and working memory.
